# No evidence tube entrapment distresses rodents in typical empathy tests

**DOI:** 10.1007/s10071-024-01863-9

**Published:** 2024-04-01

**Authors:** Dwi Atmoko Agung Nugroho, Sri Kusrohmaniah, Emma Pilz, Clare Krikorian, David Kearns, Burton Slotnick, Maria Gomez, Alan Silberberg

**Affiliations:** 1 Alumnus of Magister Psikologi, Universitas Gadjah Mada, Yogyakarta, Indonesia; 2 Fakultas Psikologi, Universitas Gadjah Mada, Yogyakarta, Indonesia; 3 Department of Psychology, American University, 4400 Massachusetts Ave., NW, Washington, DC 20016 USA

**Keywords:** Rat, Prosocial behavior, Restraint tube, Pursuit of social contact, Distress

## Abstract

In the first two experiments an empty tube open at one end was placed in different locations. Male hamsters, tested one at a time, tended to stay close to the tube or in it. During the first minute of the first 4 sessions of Experiment 3, the hamster was unrestrained. If it entered the tube, it was locked within the tube. If it did not enter the tube during the first min, it was placed in it, and the tube was locked. Fifteen min later, the tube was opened, and the hamster was unrestrained for a further 20 min. The tube remained open during Session 5. Hamsters spent more time near the tube than predicted by chance and continued to enter the tube although tube-occupancy duration did not differ from chance levels. In Experiment 4, male rats were tested in two groups: rats in one group had been previously trapped in a tube and rats in the other group allowed to freely explore the test space. For the first two min of each of four 20-min sessions, trapped-group subjects were permitted to move about the chamber unless they entered the tube. In that case, they were locked in for the remainder of the session. If, after two min, they did not enter the tube, they were locked in it for the remaining 18 min. Free rats were unrestricted in all sessions. In Session 5, when both groups were permitted to move freely in the chamber, trapped and free rats spent more time in and near the tube than predicted by chance. These data show tube restraint does not seem to distress either hamsters or rats.

## Introduction

Recently, Hachiga et al. (2020) provided evidence that locking rats in a restraint tube is not distressing. This result calls for replication because their claim has important implications for rat-empathy studies where tube-induced distress is requisite. This report is a systematic replication (Sidman 1960) of their work.

In a systematic replication, features of an experimental procedure are altered across different experimental designs to see whether a claimed empirical effect emerges when theoretically irrelevant features of design are manipulated. Rather than replicate by duplication, the intent is to replicate with variables that differ somewhat from the target study. If an effect emerges in a group of such studies, the variable is considered robust.

This approach has a long history—it defines how research is typically done in behavior analysis (Ferster and Skinner 1976). Subject numbers can be fewer and null-hypothesis statistical testing takes a subsidiary role because studies in the group must point empirically to the same conclusions. In terms of intent, such an approach shares characteristics with a meta-analysis or a concatenated *t* test: confidence in the veracity of the finding builds when results are viewed in aggregate.

### Background of this systematic replication

Ben-Ami Bartal et al. (2011) found that when a rat was trapped inside a plastic tube, a second rat that was not constrained would learn to push a door to the tube entrance aside so that the trapped rat could escape. They considered three interpretations of this result: (a) it could be an empathic act by the free rat, motivated by the desire to reduce the putative stress imposed on the trapped rat by being locked in a tube; (b) it could be the pursuit of social contact, an outcome realized by freeing the trapped rat for the value of its company; or (c) it could be the reinforcing value of tube occupancy—that is, the free rat may open the tube so that it may enjoy being in the open tube.

To select among these possibilities, Ben-Ami Bartal et al. (2011) conducted control procedures. In one, opening the front door to the tube freed the trapped rat but imposed greater distance between the freed rat and the trapped rat. They found that a free rat that had previously been trained to open the tube door continued to do so despite an outcome that they deemed incompatible with pursuing social contact. In another control procedure, experimentally naïve rats could learn to open the door to an empty tube. Their frequency of doing so was lower than that seen in rats that freed a trapped rat. Based on this difference, they concluded door opening by a free rat was not primarily attributable to an opportunity to occupy an open but empty restraint tube. By virtue of minimizing the significance of social contact and tube occupancy as reasons for opening the door to a tube, Ben-Ami Bartal et al. argued that door opening was mostly an empathically motivated action designed to reduce the distress caused by trapping a rat in the tube. They buttressed this argument by noting that some ultrasonic vocalizations in the chamber were elevated during the pre-release period in sessions with trapped rats—an outcome sometimes associated with distress in rats.

Subsequent work has questioned the adequacy of the two control procedures described above. Silberberg et al. (2014) and Silva et al. (2020) found that when experimentally naïve rats could release trapped rats to a chamber more distant from the free rat, tube opening was not maintained. If tube opening were motivated by empathic concern for the trapped rat, consistent door opening would be expected despite its escape to a distal location.

The data from the Ben-Ami Bartal et al. (2011) second control procedure were also problematic—rats opened the empty tube at a lower likelihood than the trapped-rat tube (10% vs. 77%, respectively). But why did they learn to open the tube at all? These data raise the possibility that tube entry itself may have reinforcing value. A similar result was shown in rats by Silva et al. (2020) and in mice by Ueno et al. (2019). Such outcomes seem ecologically appropriate given rats and mice are burrowing species (Boice 1977). The restraint tube might be viewed as analogous to a tunnel.

The failure of naïve rats to open a tube for trapped-rat escape to a distant chamber and the possibility that tube occupancy is reinforcing raise the importance of Ben-Ami Bartal et al. (2011) ultrasonic vocalization measure in supporting their belief that tube occupancy is distressing. Unfortunately, this measure had a checkered history as an indicant of distress prior to their report (see Portavella et al. 1993; Thomas et al. 1983); it was made more so by Kalamari et al. (2021) finding that increased alarm calling of rats was correlated with increased *inaction* in free rats during tube-based empathy tests. This result is inconsistent with the view that trapped-rat ultrasonic vocalization signals distress that results in empathic action in a free rat.

What does motivate free-rat behavior in tube-based empathy tests? Some have answered not by arguing that the data force the choice of one process vs. another (e.g., empathy vs. social contact), but by the admission of multiple processes (Kalamari et al. 2021; Silva et al. 2020). Perhaps, then, the Ben-Ami Bartal et al. (2011) result is due to one or both sociality and empathy plus, possibly, the reinforcing power of occupying an open tube. Although such an argument is unparsimonious, it is not logically flawed if the operation of multiple processes can be discerned. But to be an empathy theorist, a list of contributing factors must include empathy as a component; otherwise, one is not studying rat empathy.

Hachiga et al. (2020) directly tested whether empathy was at least one of the processes involved in tube-based rat empathy tests. In their second experiment, rats were locked into the same model restraint tube used in Ben-Ami Bartal et al. (2011). After being locked in the tube for 10 min, the door was raised, permitting egress. Once the rat was outside the tube, the door was closed. Tube re-entry required a lever response. Rats learned to press the lever to enter the tube again. The latency to press the lever decreased over sessions, an indicator that the lever press response was being strengthened.

Learning theory makes a clear prediction here: if being locked in the tube for 10 min is aversive, the rat should avoid entering the tube and perhaps even avoid the tube altogether. The fact that rats enter the tube again means that being locked in the tube is not aversive. In fact, it is reinforcing, as evidenced by the fact that the rat presses the lever for the opportunity to enter the tube. We believe this result is incompatible with the argument that rat empathy had been demonstrated by Ben-Ami Bartal et al. (2011).

In fact, we already know what would happen if a rat were truly distressed in a tube-based empathy test. Kalamari et al. (2021) note: “When we started testing, we placed the partners in a much smaller cylinder in which animals could not move or turn and showed clear physical signs of distress (such as actively struggling, excretion of feces, and urinating). In this setting, test rats did not press the lever at all, but rather *avoided* (emphasis added) the cylinder (p 12).”

To date, few have acknowledged the importance of Kalamari et al. (2021) anti-empathy finding or discussed the claims of Hachiga et al. (2020). Blystad (2021) thinks this may be due to a citation bias among empathy researchers. Of course, there is a less self-serving explanation for why studies with incompatible results are not cited: perhaps investigators are unconvinced by Hachiga et al. and Kalamari et al. results or arguments. If successful, the present systematic replication of Hachiga et al. (2020) may buttress credence in their dataset.

In the first three experiments of this systematic replication, we change the rodent species from rat to hamster to test the species generality of rat-based outcomes. Like rats, hamsters belong to Myomorpha, a suborder of Rodentia. In addition to similar morphologies (elongated body and pointed snouts), they are a burrowing species. It is this shared behavior that made selection of hamsters appealing. We argue that rats and hamsters might enter restraint tubes because they approximate the burrows they naturally create. For hamsters we used toy tubes from pet stores as restraint tubes to determine whether tube occupancy meets the criterion outcome of Hachiga et al. (2020): whether free or trapped, do hamsters find tube occupancy reinforcing? For rats we provide an independent and different systematic replication in pursuit of the same demonstration and using the same model restraint tube as in Ben-Ami Bartal et al. (2011). Across all tests the reader is reminded that for empathy for another animal’s distress to be a component of tube-based behavior in rodents, tube constraint under circumstances akin to Ben-Ami Bartal et al. must be aversive.

## Experiment 1

### Method

#### Subjects

Eight experimentally naïve male Campbell's dwarf hamsters *(Phodopus campbelli)*, 3 to 4 weeks old at the time of purchase from a local pet store, and weighing 13 to 18 g, served as subjects (Subjects 1 through 8). They were individually housed in 50 by 50 by 20-cm cages in a continuously illuminated animal colony. After one week of acclimation to the animal colony, the experiment began. The hamsters had unrestricted access to CITRAFEED Rat Bio food pellets (PT CITRA INA FEEDMILL, Jakarta, Indonesia) twice per day in their home cages. Water was freely available except during experimental sessions.

#### Apparatus

**Fig. 1:**
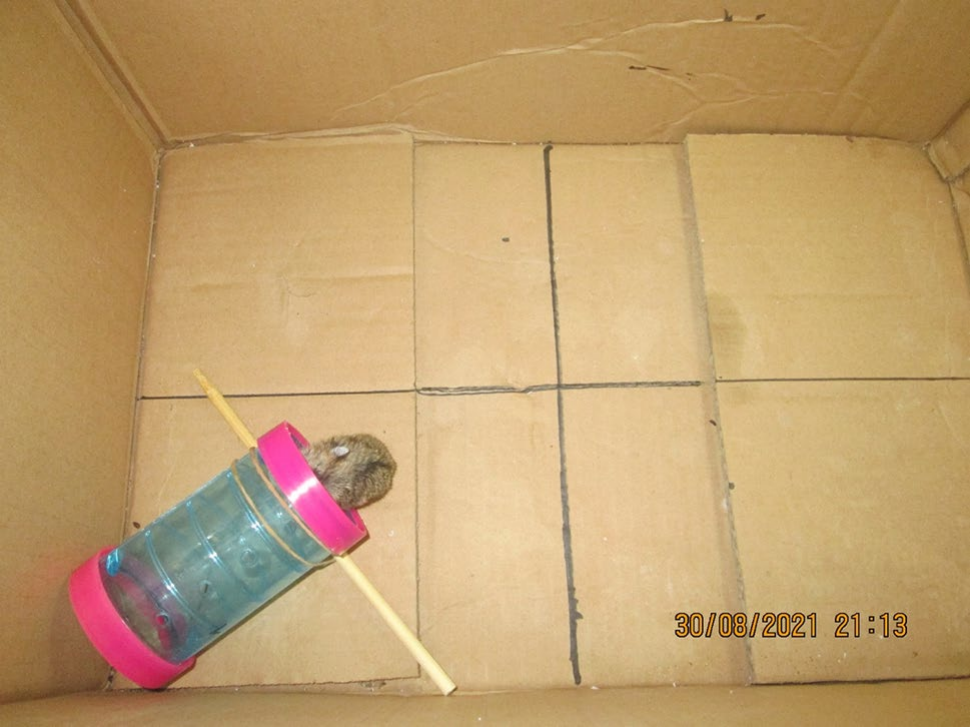
A photograph of a hamster entering a tube during a trial in Experiment 1. Note the demarcation of quadrants on the chamber floor

A 6- by 12-cm translucent acrylic tube was placed with one end in the corner of a 35- by 25- by 20-cm rectangular cardboard chamber. The other end was pointed toward the center of the chamber. A 10- by 0.3-cm stick was positioned under the opening to the tube to ensure its stability. Four chamber quadrants (Q1 through Q4) were defined by black lines drawn on the floor of the chamber (Fig. 1).

The experiment was conducted in a room sized 8 by 3 by 3 m, with a humidity level of 20%, and a temperature maintained at 23–24 °C. Room luminance levels were approximately 500 lx. All behavior was recorded by a Canon A2300 PowerShot camera operating once every 10 s.

### Procedure

During the week after the arrival of the hamsters at the experimental site, they lived in their home cages where they received twice-daily vegetable pellets, water, and access to a treadmill for their enrichment and exercise. Thereafter, the experiment began. Beginning at 7 PM, a subject was placed in the center of the experimental chamber for his daily session. Photographs of his movements in the chamber and the open tube were taken every 10 s during a 20-min session (120 photographs/session). Upon completion of the session, the hamster was returned to his home cage, the location of the tube in the chamber was altered to ensure across-subject counterbalancing of tube location, and then another subject’s session began after the tube was washed with water and dried. This regimen continued until all subjects had completed their session. Five daily 20-min sessions defined the experiment. Due to experimenter error, measures of latency to first entry of a tube are unavailable in this experiment as well as Experiments 2 and 3.

### Results

Table 1 presents the frequency data for each subject in terms of the quadrant occupied at the time of photograph and whether it was inside the tube or not. In the event the hamster spanned two quadrants, the experimenter judged which quadrant contained more of the subject’s body and categorized that quadrant as the one occupied. A similar rule was used to define whether a subject was inside the tube or not. Tallies of tube occupancy did not also count as instances of quadrant occupancy. As shown by the means in the bottom row of the Table, the hamsters spent more of the session in the quadrant containing the tube than would be predicted by chance. However, they spent more time outside the tube than inside it.

**Table 1:** Results of experiment 1

(1) Subject (tube quadrant)	Photos/session of subject location (120 observations)		Photos/session (120 observations)	
	(2) Tube quadrant	(3) Non-tube quadrant	(4) Subject inside tube	(5) Subject outside tube
1 (Q1)	99	21	47	73
2 (Q2)	100	20	32	88
3 (Q3)	84	36	40	80
4 (Q4)	81	39	36	84
5 (Q1)	97	23	53	67
6 (Q2)	101	19	44	76
7 (Q3)	103	17	64	56
8 (Q4)	96	24	72	48
Mean (SD)	95.125 (8.1)	24.875 (8.1)	48.5 (13.8)	71.5 (13.8)

The subject number and quadrant of the tube location in the last session (Column 1); frequency photos showed the number of times the subject was in tube quadrant (Column 2) vs. not in that quadrant (Column 3); frequency photos also show when subject was inside the tube (Column 4) vs. outside (Column 5)

Of course, these calculations ignore the fact that the quadrant containing the tube and the tube itself respectively occupied only 0.25 and 0.082 of the surface of the chamber. If one makes the simplifying assumption that in the absence of a tube, a hamster’s location would be randomly distributed throughout the chamber, 0.25 × 120 = 30 photographs would be expected to show the hamster in the tube quadrant and 0.082 × 120 = 9.8 photographs would be expected to show the hamster occupying the tube. Instead, a mean of 95.1 photographs showed the subject in the tube quadrant and a mean of 48.5 photographs showed the subject occupying the tube. A two-tailed one-sample Wilcoxon signed-ranks test found that there were significantly more photographs of the hamster in the quadrant containing the tube than would be expected if location were randomly distributed (*T*^+^(8) = 36, *p* < 0.01). A similar analysis found that the number of photographs with the hamster in the tube was also significantly greater than would be expected if the subjects’ locations were randomly distributed (*T*^+^(8) = 36, *p* < 0.01).

**Fig. 2:**
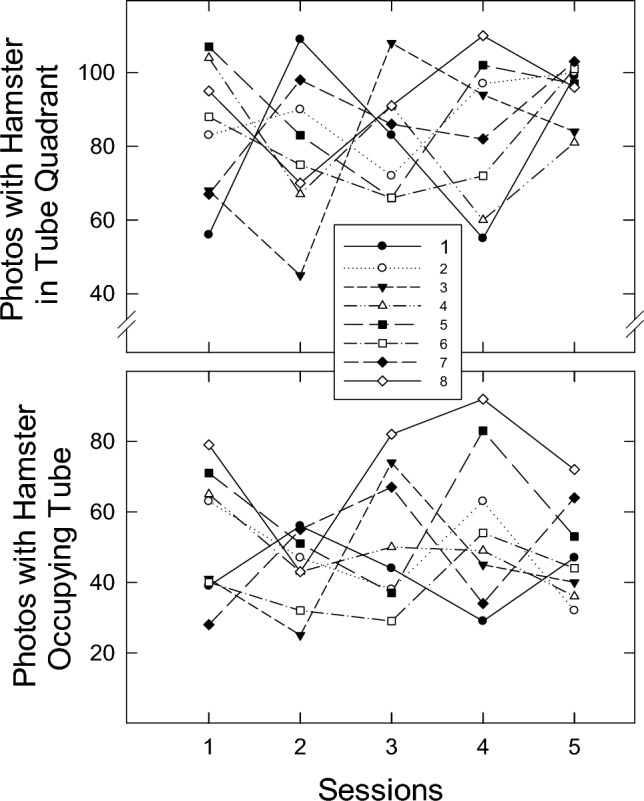
Individual-subject data for all sessions based on photographs taken every 10 s. The top panel presents the frequency with which the hamster was in the quadrant containing the tube, but not in the tube itself. The bottom panel presents the number of times the hamster was in the tube

One-hundred-twenty photographs were taken of each hamster’s position in the chamber during each of the five sessions of Experiment 1. The top panel of Fig. 2 presents the number of photographs in which a subject was in the chamber quadrant containing the tube based on experimenter judgment. The bottom panel reflects the frequency with which a subject was judged to be inside the tube. No across-session trends are apparent in either data set (Friedman’s tests: both *χ*^2^(4)s ≤ 4.5, both *p*s > 0.30).

### Discussion

In this experiment, the hamsters tended to be near the tube, regardless of the quadrant in which the tube was placed, and they also tended to be often in the tube itself. Since there is little evidence of changes in tube proximity and occupancy across sessions, there is little reason to suspect that these results are due to learning. Indeed, it seemed likely that tube occupancy was naturally appealing to the subjects from the start. As hamsters live in underground tunnel and nest systems, it is likely that the tube provided an ecologically acceptable substitute for the tunnels that they naturally use.

## Experiment 2

In the prior experiment, we compared photographs of a hamster’s proximity and occupancy of the tube against that of a hypothetical responder who assumed a randomly chosen location in the chamber. While this hypothesis simplified data analysis, there is reason to doubt hamsters would position themselves at random in a chamber without a tube. Rats are thigmotactic (e.g., Barnett 1975; Lamprea et al. 2008), a propensity that causes them to stay close to walls when first encountering a new arena. Assuming that hamsters are similarly disposed (e.g., Bastida et al. 2009), hamster proximity to the tube might have been favored not because the tube is natively appealing, but because it was placed in the corner of the chamber where adjacent walls meet. To test for this possibility, Experiment 2 repeats the design of the prior study with the tube placed not in a corner of the chamber, but at its center. If thigmotaxis contributed to the significant results in Experiment 1, it should impede replication in Experiment 2.

### Method

#### Subjects

Eight experimentally naïve male Campbell's dwarf hamsters *(Phodopus campbelli)*, 3 to 4 weeks old at arrival to the laboratory, and weighing 13 to 18 g, served as subjects (Subjects 9 through 16). Their maintenance and housing conditions were unchanged from those present in Experiment 1.

#### Apparatus

The apparatus was the same as in Experiment 1 except for the fact that a plastic cap was placed over one end of the tube. In other respects, the apparatus was unchanged from Experiment 1.

#### Procedure

**Fig. 3:**
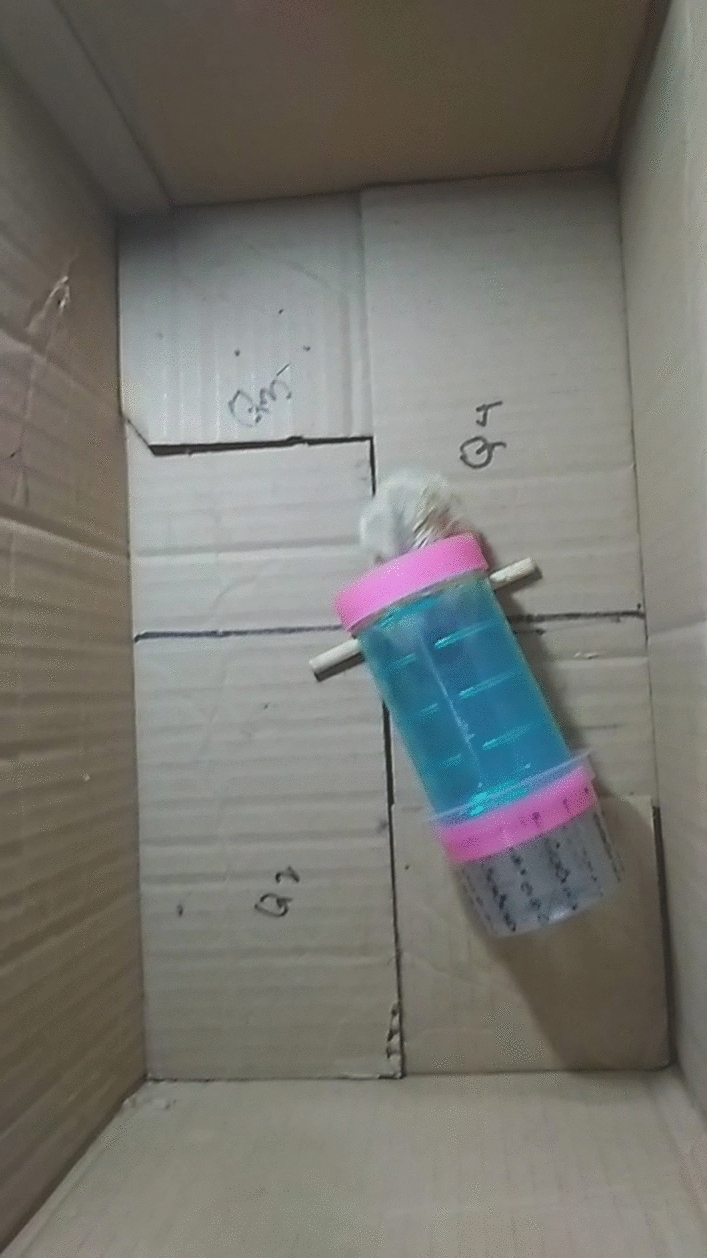
A photograph of a hamster entering a tube during a trial in Experiment 2

As shown in Fig. 3, the open end of the tube was positioned in the center of the chamber with the closed end pointed toward one of the four corners of the chamber. The orientation of the tube was counterbalanced across subjects so that each corner of the chamber was equally likely to have the closed end of the tube facing it. In other respects, the procedure was unchanged from that used in Experiment 1.

### Results and discussion

Table 2 presents the frequency with which hamsters were in the quadrant containing the tube and in the tube itself during the fifth session of the experiment. These results appear similar to those from Experiment 1. There was no significant difference across experiments for either the quadrant-location test (two-tailed Mann Whitney test: *U*(8,8) = 28, *p* > 0.7) or the tube-occupancy test (Mann Whitney *U*(8,8) = 23, *p* > 0.35). Additionally, in Experiment 2 photographs showed that hamsters were in the tube quadrant and in the tube itself significantly more often than would be expected had their locations been randomly distributed (two-tailed one-sample Wilcoxon signed-ranks tests: *T*^+^(8) = 36, *p* < 0.01, for both comparisons). Given these results it seems safe to conclude that hamsters prefer to be proximal to, and inside of tubes (as had been shown in Experiment 1), and that this tendency is largely independent of the location of the tube in the chamber.

**Table 2:** Results of experiment 2

(1) Subject (tube quadrant)	Photos/session of subject location (120 observations)		Photos/session (120 observations)	
	(2) Tube quadrant	(3) Non-tube quadrant	(4) Subject inside tube	(5) Subject outside tube
9 (Q1)	110	10	65	55
10 (Q2)	88	32	29	91
11 (Q3)	116	4	28	92
12(Q4)	106	14	95	25
13 (Q1)	93	27	20	100
14 (Q2)	102	18	25	95
15 (Q3)	39	81	16	104
16 (Q4)	82	38	73	47
Mean (SD)	92 (24.3)	28 (24.3)	43.875 (29.5)	76.125 (29.5)

Subject number and quadrant of tube location in last session (Column 1); frequency of photos showing subject in tube quadrant (Column 2) vs. not (Column 3); frequency of photos showing subject inside tube (Column 4) vs. outside (Column 5)

## Experiment 3

Based on the data of the first two experiments, hamsters tend to occupy the tube when one side was open. They might not have done so had they been locked into the tube as was done to the trapped rats in Ben-Ami Bartal et al. (2011). Hachiga et al. (2020) found in the second phase of their second experiment that rats that had been locked in a restraint tube for 10 min prior to release subsequently pressed a lever to re-enter that tube. Rather than finding 10 min of entrapment distressing, it appears from their data that rats found tube restraint rewarding. The present experiment is a systematic replication of their research using hamsters instead of rats.

Individual hamsters were put in the chamber with a restraint tube open at one end. If, within the first min, the hamster entered the tube, a cap was placed over the open end, thus trapping the animal. If the hamster did not enter the tube during this interval, it was placed in the tube by the experimenter thus trapping it therein. Whether trapped because of its own action or by the experimenter’s, the cap was removed after 15 min and the hamster’s location in the chamber was photographed every 10 s for the next 20 min. Based on the findings of Hachiga et al. (2020), we expected hamsters to re-enter the tube despite prior entrapment.

### Method

#### Subjects

Eight experimentally naïve male Campbell's dwarf hamsters *(Phodopus campbelli)*, 3 to 4 weeks old upon arrival at the laboratory, and weighing 13 to 18 g, served as subjects (Subjects 17 through 24). Their maintenance and housing conditions were unchanged from those used in Experiment 1 and 2.

#### Apparatus

The apparatus was the same as in Experiment 1. However, a plastic cap was placed over one end of the tube and an identical cap was available to place over the other end of the tube during the experiment. In other respects, the apparatus was unchanged from Experiment 1and 2.

#### Procedure

The tube was placed in the center of the chamber, and its orientation counterbalanced across subjects in the manner described in Experiment 2. During the first four sessions, the end of the tube more distal to the chamber’s center was covered by a plastic cap, the other end being left open. A session began when a single hamster was placed in the chamber. If, during the first min, it entered the tube, the open end of the tube was covered with a plastic cap. If the hamster did not enter during the first min, it was placed in the tube and the second cap was used to lock the hamster inside. After 15 min, the recently added cap was removed, and the hamster’s location was photographed every 10 s for the remaining 20 min of the session. In Session 5, the hamster was free to move about the chamber for 20 min without any experimenter intervention. The previously capped end of the tube was left open. In other respects, the procedure was unchanged from Experiment 2.

### Results and discussion

Session 5 was procedurally identical in Experiments 2 and 3 in that one end of the restraint tube was open throughout the session for 20 min. Experiment 2 differed from Experiment 3 in that the tube was also open during Sessions 1 through 4 but closed in Experiment 3. This arrangement permits determining how a history of tube restraint (Experiment 3) affects Session-5 quadrant selection and tube-entry duration relative to hamsters that were not restrained in Sessions 1–4 of Experiment 2. Using Session-5 results presented in Table 2 (Experiment 2) and Table 3 (Experiment 3), we made two, two-tailed Mann Whitney tests to compare these measures. For the test of hamster proximity to the tube, *U*(8,8) = 7.5, *p* < 0.05; for the test of duration of occupancy of a tube, *U*(8,8) = 0, *p* < 0.001. As is also clear from inspection of the Tables, a four-session history of tube restraint in Experiment 3 significantly reduced the likelihood of proximity to the tube and tube entry. Despite this fact, a history of tube restraint did not make hamsters avoid tube occupancy or proximity below levels that would be expected by random location in the chamber. They were photographed a mean of 57.3 times in the tube quadrant, which was significantly greater than the 30.0 photographs that would be expected to show such occupancy if location had been randomly determined (two-tailed one-sample Wilcoxon signed-ranks test: *T*^+^(8) = 36, *p* < 0.01). In terms of tube occupancy, 9.8 photographs were expected, based on chance to show such occupancy vs. the 8.0 such photographs obtained. This result was, however, not significant (two-tailed one-sample Wilcoxon signed-ranks test: *T*^+^(8) = 6, *p* > 0.1). These results are consistent with those of Hachiga et al. (2020) in demonstrating that tube occupancy is not distressing to subjects.

**Table 3:** Results of experiment 3

(1) Subject & tube quadrant	(2) Tube location in chamber	Photos/session of hamster in the tube’s quadrant following release				
		Exit blocked				Exit open
		Session 1	Session 2	Session 3	Session 4	Session 5
17 (Q1)	Center	59	20	17	56	73
18 (Q2)	Center	67	45	77	23	82
19 (Q3)	Center	87	53	45	9	57
20 (Q4)	Center	40	66	21	61	43
21(Q1)	Corner	91	50	66	49	45
22 (Q2)	Corner	32	26	39	11	34
23 (Q3)	Corner	66	41	31	20	56
24 (Q4)	Corner	12	32	52	17	68
Mean (SD)		56.75 (27.2)	41.625 (15.2)	43.5 (21.0)	30.75 (21.1)	57.25 (16.4)

Subject & tube quadrant	Tube location in chamber	Photos/session with hamster in the tube following release				
		Exit blocked				Exit open
		Session 1	Session 2	Session 3	Session 4	Session 5
17 (Q1)	Center	1	2	1	3	12
18 (Q2)	Center	2	2	3	2	7
19 (Q3)	Center	1	2	4	2	11
20 (Q4)	Center	1	1	1	1	9
21(Q1)	Corner	1	2	1	2	6
22 (Q2)	Corner	1	2	3	2	8
23 (Q3)	Corner	1	1	2	1	6
24 (Q4)	Corner	2	2	1	3	5
Mean (SD)		1.25 (0.46)	1.75 (0.46)	2.00 (1.20)	2.00 (0.76)	8.00 (2.51)

For all panels, subject number and quadrant of tube (Column 1); center or corner location of tube within quadrant (Column 2); remaining columns present data for each session. In the first four sessions, the hamster was locked in the tube at the session’s start and then released. In Session 5, the door was not used to block egress. The top panel presents the photos/session of the hamster in the tube quadrant after release (Sessions 1 to 4) or for the entire session (Session 5). The bottom panel presents how often the hamster entered the tube after release

In Experiment 3, there was no significant trend over the first four sessions, where the hamster was trapped inside the tube prior to being freed, for either the time-near-the-tube or the time-in-the-tube measure (Friedman’s tests: both *χ*^2^(4)s ≤ 5.7, both *p*s > 0.05). When comparing session 5, where the hamster was never trapped, to session 4 there was no significant difference in time near the tube (Wilcoxon signed-ranks test: *T*^+^(8) = 32, *p* > 0.05). For the time-in-the-tube measure, there was a significant increase from session 4 to session 5 (Wilcoxon signed-ranks test: *T*^+^(8) = 36, *p* < 0.01). This suggests that hamsters are more likely to enter the tube when not having just spent the previous 15 min inside of it.

Despite affirmation by data from Hachiga et al. (2020), theorists focused on empathy might argue that the reduction of tube occupancy time seen in hamsters in Experiment 3 relative to Experiment 2 was due to the growth of restraint-induced distress. Such an account would seem to argue that the net effect of increasing distress was to reduce the reinforcing power of tube occupancy. A learning theorist might counter that this result might be not due to distress, but rather to habituation. In other words, perhaps tube restraint did not cause distress, but it might have caused hamsters to become bored of being in the tube. It is also worth noting that a similar effect was seen in Hachiga et al. (2020), although in their case the measure was mean time in the tube. Across different publications, experimental designs, and subject populations, there is evidence that repeated exposure to a tube diminishes the reinforcing power of tube occupancy for a rodent.

## Experiment 4

The results up to this point show that for hamsters, there is no evidence that occupancy of a tube was aversive even when tube egress was prevented. This was the same result reported by Hachiga et al. (2020). Still, it is possible that this apparently analogous finding might be due to different psychological processes because of differences between hamsters and rats. To address this possibility, we replicated the procedures presented earlier, this time with rats as subjects. In keeping with the intent of systematic replication, this design differs somewhat from those in the hamster experiments in this report and in Hachiga et al. (2020). If the results we have reported are robust, these procedural changes should have little effect on outcomes.

### Method

#### Subjects

Sixteen female Long Evans rats (Subjects 25–40), between 9 and 10 weeks of age, weighing approximately 250 g served as subjects. The rats were purchased from Envigo, Livermore, CA. All rats were previously subjects in an unrelated experiment that involved lever pressing for saccharin reinforcers in an operant chamber. The rats were individually housed in plastic cages located in an animal colony room with a 12-h light–dark cycle with the light phase beginning at 8 AM. Sessions took place during the light phase. Rats had free access to food and water in their home cages throughout the experiment.

#### Apparatus

Sessions took place in a small testing room in which illumination was provided by a 72-w light bulb. A 50-gallon plastic tub (Sterlite, Townsend, MA), measuring 108.6 cm long, 55.9 cm wide and 45.7 cm high was placed on the floor. The same model Harvard Apparatus (Holliston, MA) restraint tube that Ben-Ami Bartal et al. (2011) used was used here. It was made of clear acrylic and measured approximately 25 cm in length with a diameter of 8.5 cm at its widest point. The tube was mounted on a clear acrylic platform approximately 1.5 cm high. Clear acrylic doors could be placed at the front and rear of the tube to block egress. The rear door was glued in place approximately 2.5 cm from the rear of the tube. The front door could be inserted into and removed from one of several slots. In the present experiment, when the front door was inserted, the length of the interior of the tube measured from the inside edge of the front door to the inside edge of the back door was 17 cm. When a rat was trapped in the tube, the front door was secured in place with two pieces of electrical tape. Sessions were videorecorded by a Logitech C270 (Logitech, Lausanne, Switzerland) camera that was connected to a laptop computer. The apparatus was cleaned with Cetylcide II after each experimental session and whenever a prior subject soiled the restraint tube.

#### Procedure

**Fig. 4:**
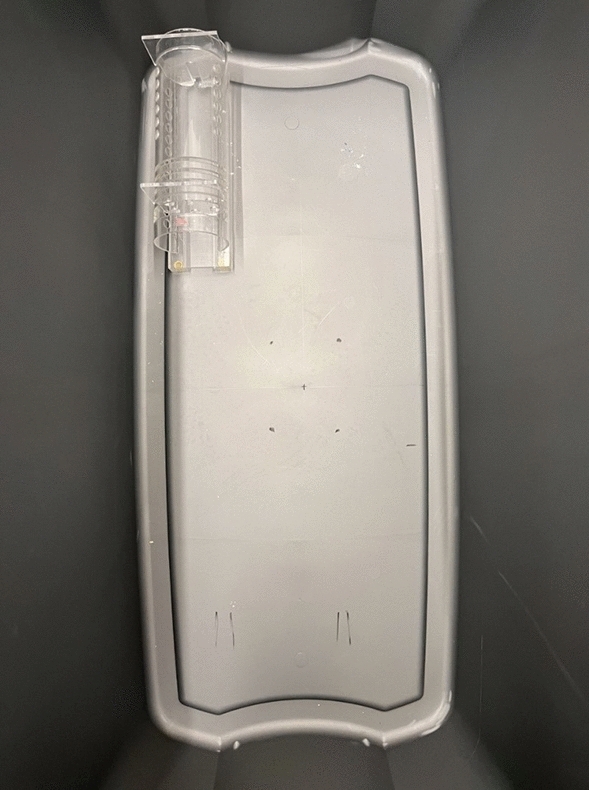
Photograph taken from above of the bin with the tube in one of the corners in Experiment 4

Prior to the start of the experiment, rats were randomly assigned to Trapped (*n* = 8) or Free Groups (*n* = 8). The tube was placed in one of the four corners of the tub such that the tube was parallel to one of the long walls of the tub and the rear end of the tube was near one of the shorter walls of the tub. Each of the four corners of the tub was used; tube placement in a corner was counterbalanced over rats in each group. For each rat, the tube was always placed in the same corner across sessions. Figure 4 is a photograph of the apparatus inside the bin.

The experiment consisted of several training sessions and a final test session. The two groups differed with respect to the procedure used for the training sessions. For rats in the Trapped Group, the experimenter began a training session by placing the subject in the center of the bin. If the rat entered the tube within 2 min, the front door was secured in place and the rat was trapped in the tube for the remainder of the 20-min session. If the rat had not entered the tube by the 2-min mark, the experimenter gently coaxed the rat into the tube and then secured the front door, trapping the rat in the tube for 18 min. For rats in the Free Group, training sessions began with the experimenter placing the rat in the center of the tub. These rats were permitted to explore the tub and the open tube for a 20-min session. The front door was never inserted at any time for rats in the Free Group. At the end of training sessions, the rat was removed from the bin and returned to its home cage. The original plan had been to give rats in each group four training sessions before the final test. Due to experimenter error, half of the rats in each group had four sessions and the other half had five sessions prior to the test. On the test session, rats in both groups were allowed to explore the bin and the open tube for 20 min. This meant that the test session was identical to training sessions for the Free Group.

The primary measure was where rats spent their time during the session. Videos were analyzed to obtain measures of total time in the tube and in the third of the bin containing the tube for each rat. Additionally, the latency to enter the tube during the first 2 min of the session on the first training session and the test session was measured. Only the first 2 min were analyzed because rats in the Trapped Group were placed into the tube after 2 min if they had not already entered on their own. If a rat in either group did not enter the tube on its own within 2 min, it was assigned the maximum latency of 2 min.

During the test session, the groups were compared with respect to the percentage of time spent in the tube and in the third of the chamber containing the tube using two-tailed Mann Whitney tests. Additionally, to test whether rats spent more or less time in the tube than would be expected by chance, two-tailed, one-sample Wilcoxon signed-ranks tests were used to compare the percentage of total time spent by each group in the tube to 6.6%, which corresponds to the floor area taken up by the tube. Similarly, one-sample Wilcoxon signed-ranks tests were used to compare the percentage of total time spent by each group in the third of the chamber containing the tube to 33.3%. For the latency data, Wilcoxon signed-ranks tests were performed on the groups’ latencies to enter the tube on the first session and the test session. Additionally, between-group comparisons were made of latencies during these sessions with Mann Whitney tests.

### Results

**Fig. 5:**
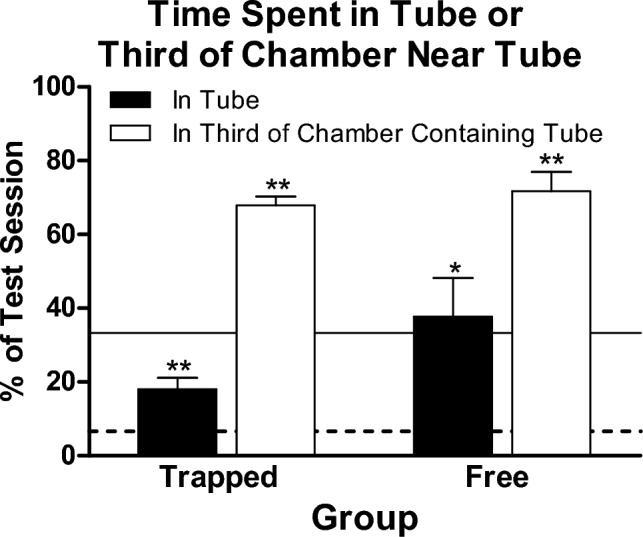
Mean (± SEM) percentage of time spent in the tube (white bars) and in the third of the chamber black bars) containing the tube during the test session for the Trapped and Free Groups. The solid horizontal line represents 33.3%, the percentage of time expected to be spent in the third of the chamber containing the tube if rats’ locations were randomly distributed. The dashed horizontal line represents 6.6%, the percentage of time expected to be in the tube if rats’ locations were randomly distributed. * indicates *p* < 0.05, ** indicates *p* < 0.01

Figure 5 shows for both groups the mean percentage of time during the test session spent in the tube and in the third of the bin containing the tube. Rats in the Trapped and Free Groups spent approximately 20% and 40%, respectively, of the test session in the tube. The difference between groups was not significant (Mann Whitney test: *U*(8,8) = 17, *p* > 0.1). For both groups, the time spent in the tube was significantly greater than 6.6%, which is the percentage expected if rats’ location during the test were randomly distributed (two-tailed, one-sample Wilcoxon signed-ranks tests: Trapped group, *T*^+^(8) = 36, *p* < 0.01; Free Group, *T*^+^(8) = 35, *p* < 0.05). A similar picture emerged for the time spent in the third of the chamber containing the tube. Both groups spent approximately 70% of session time in that third of the bin (no group difference, Mann Whitney test: *U*(8,8) = 25, *p* > 0.5), which is about twice what would be expected if rats’ locations were randomly distributed. For both groups, the time spent in the third of the bin near the tube was significantly greater than 33.3% (for both groups, two-tailed one-sample Wilcoxon-signed ranks tests: *T*^+^(8)s = 36, *p*s < 0.01). Table 4 presents the individual-subject data from which this Figure is composed.

**Table 4:** Time and location of rats in experiment 4

Trapped group			Free group		
Subject	Time (s) in tube	Time (s) in third of chamber containing tube	Subject	Time (s) in tube	Time (s) in third of chamber containing tube
26	17.8	64.1	25	20.7	64.3
27	10.8	63.8	28	79.6	91.7
29	10.0	61.3	31	26.1	59.1
30	17.4	62.9	32	64.2	85.0
34	17.3	78.9	33	19.7	67.8
36	17.0	68.3	35	5.8	49.3
37	15.3	65.2	38	12.3	69.8
39	38.4	78.3	40	73.5	86.8
Mean (SD)	18.0 (8.8)	67.8 (6.9)	Mean	37.7 (29.6)	71.7 (14.8)

Mean and individual subjects’ time spent in the tube and in the third of the chamber containing the tube during the test session in Experiment 4

**Fig. 6:**
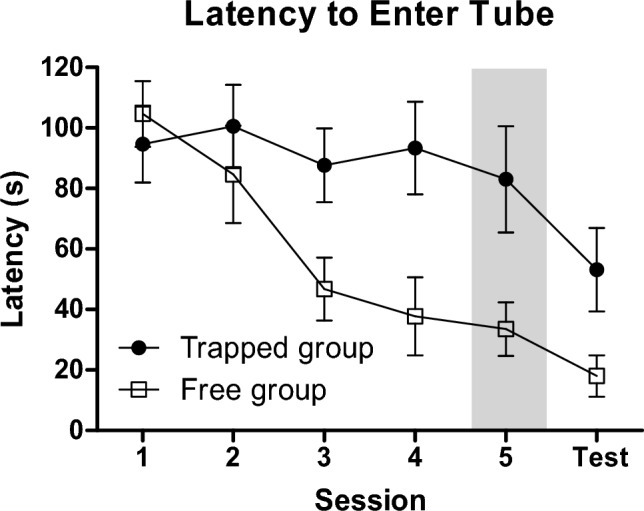
Mean (± SEM) latencies (s) to enter the tube during the first two minutes of a session across training and test sessions for the Trapped Group (circles) and the Free Group (squares). Session 5 is shaded in gray to indicate that only half the subjects in each group had a fifth training session prior to the test. *** indicates *p* < 0.001 for the main effect of Session for the comparison of Session 1 and the test session

Figure 6 shows that for the Trapped Group, the mean latency to enter the tube on the test session was approximately half the latency to enter the tube in the first session. For the Free Group, the mean latency to enter the tube on the test session was about one fifth that observed in the first session. Two-tailed Wilcoxon signed-ranks tests found that the decrease in latencies from the first session to the test session was significant for the Free Group (*T*^+^(8) = 36, *p* < 0.01), but did not reach significance for the Trapped Group (*T*^+^(8) = 29, *p* > 0.1). Two-tailed Mann Whitney tests found that latency did not differ by group on the first test session (*U*(8,8) = 27, *p* > 0.6), but the Free group had significantly shorter latencies on the test session (*U*(8,8) = 13, *p* < 0.05). The across-session performances defining these mean values are presented in Table 5.

**Table 5:** Latencies to tube entry in experiment 4

Subject	Session 1	Session 2	Session 3	Session 4	Session 5	Test session
Trapped group						
26	120	120	74	120	38	33
27	65	120	120	120	99	4
29	120	120	120	113	120	57
30	120	69	73	12	75	48
34	36	120	19	106	–	120
36	56	120	95	120	–	84
37	120	15	80	37	–	71
39	120	120	120	119	–	8
Mean (SD)	94.6 (35.9)	100.5 (38.9)	87.6 (34.6)	93.4 (43.3)	83.0 (35.2)	53.1 (39.0)
Free group						
25	120	120	56	57	45	53
28	37	120	15	34	13	3
31	80	73	9	30	25	41
32	120	91	43	18	51	10
33	120	32	68	16	–	12
35	120	118	94	120	–	21
38	120	120	65	11	–	2
40	120	3	24	16	–	2
Mean (SD)	104.6 (30.7)	84.6 (45.5)	46.8 (29.4)	37.8 (36.4)	33.5 (17.6)	18.0 (19.3)

Mean and individual subjects’ latencies in s to enter tube during first two min of each session and the test session. Half of the subjects in each group inadvertently had five sessions rather than the planned four, which is why there are dashes rather than numbers for Session 5 for half the rats

### Discussion

The core empirical effects reported by Hachiga et al. (2020) were replicated in Experiment 4: (a) rats spent more time proximal to the restraint tube and actually in the restraint tube, during the last session of the study, whether they had previously been trapped in the tube or not; and (b) the latency of first entry into the tube did not increase across sessions in either the trapped or free condition, but instead mean latency was numerically lower on the test session than session 1 in both conditions; however, this difference reached statistical significance only in the free condition. These results were obtained even though the two-experiment design of Hachiga et al. (2020) was changed to a single, five-session procedure in the present research. Additional between-study differences were whether a lever press was used, the experimental history, the sex, the breed and the age of the rats. Despite these differences, the results of this experiment look similar to those obtained by Hachiga et al. (2020). Most importantly, in Experiment 4 and in the research reported by Hachiga et al. (2020), regardless of whether rats had been trapped in a tube or had been free to move in and out of the tube, the latency to entry did not increase over sessions. These results indicate that tube occupancy was not aversive even if rats had been previously locked in the tube. These results contradict the view held by Ben-Ami Bartal et al. (2011) that being locked in a restraint tube is distressing to rats.

## General discussion

The results of the present studies fail to support the contention of Ben-Ami Bartal et al. (2011) that confinement in a restraint tube is distressing. Indeed, our results suggest that rats and hamsters may find entering a “restraint” tube rewarding. These outcomes are in accord with Hachiga et al. (2020) demonstration that rats that had previously been restrained would later learn to press a bar to gain ingress to that same tube. Earlier, Silberberg et al. (2014) faulted the claim by Ben-Ami Bartal et al. (2011) that they had demonstrated rat empathy based on evidence that their sociality-control condition fails when free rats are experimentally naïve (see Hiura et al. 2018; Silva et al. 2020). Subsequent work in which empathy and social-contact accounts have been compared has supported the primacy of a social process in explaining tube-based behavior (Hachiga et al. 2018; Heslin and Brown 2021). As we and Blystad (2021) note, these results have generally not been addressed in reports favoring evidence for empathy as a motivating force using tube-based release behavior by free rats.

We advanced two possibilities as to why pursuit of social contact has not impressed rat-empathy theorists who work with tube-restraint tasks. First, a risk in all research is that perhaps this work seems to readers methodologically inadequate. Rather than announce the inadequacies, empathy theorists may have just ignored them and the papers in which they were presented. A second possibility seems to us more credible: perhaps empathy theorists believe pursuit of social contact is a reinforcer for rats and may contribute to tube-opening behavior, but it is only one of several processes in operation, one of which is empathy.

Silberberg et al. (2014) argued against this multi-process view. They noted that if one rejects the adequacy of Ben-Ami Bartal et al. (2011) social-contact control, the other research from their paper as well as that of Silberberg et al. and Hachiga et al. (2018) can be accommodated in terms of sociality. If so, the principles of parsimony and Morgan’s Canon argue that a social-contact interpretation is to be preferred.

Correct or not, this argument has not guided research on tube-based rat empathy. Multi-process accounts have been advanced in which empathy is a component (Kalamari et al. 2021; Silva et al. 2020). There are no logical grounds why other factors cannot contribute to the reinforcing power of empathically motivated action. Such a step accommodates Silberberg et al. (2014) and Hachiga et al. (2018) while preserving empathy as process in rat-empathy tube tests. It is for this reason that the results of Hachiga et al. (2020) are important, for they remove empathy and social contact as explanations for opening and entering a tube because, in that study, there is no other rat to rescue or to socialize with. Hachiga et al. (2020) asked a question for which empathy accounts require an affirmative answer: is restriction to a tube like those used by Ben-Ami Bartal et al. (2011) aversive? Hachiga et al. (2020) found the answer to be “no”. In fact, in that study, rats readily entered the tube both before and after being confined in it.

The results of the Hachiga et al. (2020) study together with the present findings argue strongly that the type of restriction used by Ben-Ami Bartal et al. (2011) is not aversive and, in fact, provides a pleasant environment for the trapped rat, one to which it is willing to return again and again, and to work to enter. These are hallmarks of positive reinforcement, and they do not support claims that tube restriction causes distress in a trapped rat, motivating an empathic reaction in a free rat. Despite this fact, it must be remembered that weakening confidence in the validity of the tube-restraint procedure as a means of assessing rat empathy makes no statement about whether rat empathy exists. Indeed, it is possible that rat empathy or, at least evidence for altruistic behavior, can emerge in other kinds of tests.

## Data Availability

All quantitative data associated with this manuscript are presented in Tables and Figures. Photographs in Experiments 1, 2 and 3 are available on request from D. K. Photographs in Experiment 4 are available on request from D. K.
